# Distribution of two rare taxa of caddisflies (Trichoptera: Rhyacophilidae, Polycentropodidae) from the Republic of Kosovo

**DOI:** 10.3897/BDJ.7.e46466

**Published:** 2019-12-13

**Authors:** Halil Ibrahimi, Ruzhdi Kuçi, Astrit Bilalli, Milaim Musliu, Arben Gashi, Naman Sinani, Besnik Emërllahu

**Affiliations:** 1 Department of Biology, Faculty of Mathematics and Natural Sciences, University of Prishtina "Hasan Prishtina", Prishtina, Kosovo Department of Biology, Faculty of Mathematics and Natural Sciences, University of Prishtina "Hasan Prishtina" Prishtina Kosovo; 2 Faculty of Education, University of Prishtina "Hasan Prishtina", Prishtina, Kosovo Faculty of Education, University of Prishtina "Hasan Prishtina" Prishtina Kosovo

**Keywords:** Aquatic insects, rare species, *Polycentropus ierapetra slovenica*, *Rhyacophila aurata*, Balkans.

## Abstract

**Background:**

The knowledge about distribution, ecology and species composition of caddisflies of the Balkan Peninsula is still not complete. The ongoing investigations of the last years highlight this area as an important hotspot of caddisfly diversity. *Polycentropus ierapetra slovenica* has been considered a narrow range endemic of Slovenia and surrounding areas. *Rhyacophila aurata*, a species known from many parts of Europe, according to the current knowledge, is absent from a large part of the Balkan Peninsula.

**New information:**

In this paper, we present records of these two rare taxa of Trichoptera from the Republic of Kosovo with exact distribution data, based on sampling carried out randomly during 2014 and 2017. *Polycentropus ierapetra slovenica* was found in several streams in Bjeshkët e Nemuna Mountains and Karadak Mountains. *Rhyacophila aurata* was found during this investigation at a single locality in Bjeshkët e Nemuna Mountains.

The unexpected finding of these two taxa in Kosovo greatly enlarges their known distribution area and makes a contribution towards the better knowledge of distributional patterns of these rare taxa of caddisflies in this part of Europe.

## Introduction

The knowledge about distribution, ecology and species composition of caddisflies in the Balkan Peninsula is still not complete. The recent and ongoing investigations in this area (e.g. Bilalli et al. 2018, Gashi et al. 2014, Ibrahimi and Vehapi 2017, Oláh et al. 2018, Previšić et al. 2014, Rimcheska et al. 2015, Waringer et al. 2015) make possible the better understanding of distribution patterns and ecological features and consequently increase conservation efforts for these species and their associated habitats.

Until recently, there were only few registered records of caddisfly species in Kosovo, but the list has significantly increased during the past decade, with several new species being described (e.g. Gashi et al. 2014, Ibrahimi and Vehapi 2017, Ibrahimi et al. 2014, Ibrahimi et al. 2018, Ibrahimi et al. 2012, Ibrahimi et al. 2015, Ibrahimi et al. 2016, Karaouzas et al. 2018). However, some areas, especially the high altitude mountains of Bjeshkët e Nemuna, Sharr, Karadak and Kopaonik, have still not been fully investigated. Caddisflies of large lowland rivers and downstream freshwater ecosystems are also not very well known.

The main goal of this study was to contribute to the list of caddisfly taxa in the Republic of Kosovo and improve the knowledge about geographic distribution of rare taxa of caddisflies in the Balkan Peninsula, to assist in proper conservation of freshwater ecosystems.

## Materials and methods

Sampling was carried out occasionally during 2014 and 2017 at six sampling stations, three of them being located in Bjeshkët e Nemuna and the other three in Karadak Mountains in the Republic of Kosovo (Table 1). Adult caddisflies were collected by using ultraviolet light traps during the night and entomological nets during the day. UV light traps (according to Malicky 2004) were placed on stream banks immediately after dusk and operated throughout the night. Collected samples were preserved in 75% ethanol. The specimens were identified under a stereomicroscope using appropriate keys (Kumanski 1985, Kumanski 1988, Malicky 2004). Systematic presentation follows Morse (Morse 2019).

## Taxon treatments

### Polycentropus ierapetra slovenica

Malicky, 1998

F78BD300-7078-52F6-A6D0-77ECB1B42750

https://www.gbif.org/species/6262227

#### Materials

- **Type status:** Other material. **Occurrence:** recordedBy: Halil Ibrahimi, Ruzhdi Kuçi, Astrit Bilalli, Milaim Musliu; individualCount: 5; sex: males; **Location:** higherGeography: Europe; waterBody: tributary of Lepenc River, Aegean Sea Basi; country: Kosovo; municipality: Hani i Elezit; locality: Dërmjak; verbatimLocality: streamlet above the village, towards the border with the Republic of North Macedonia; decimalLatitude: 42.17264; decimalLongitude: 21.31582; **Event:** samplingProtocol: UV light trap; samplingEffort: overnight; year: 2017; month: 6; day: 12; fieldNotes: collected with ultraviolet light over the white pan operating from dusk until the next morning; **Record Level:** institutionCode: University of Prishtina "Hasan Prishtina", Faculty of Mathematics and Natural Sciences, Department of Biology; collectionCode: caddisflies of Karadak
- **Type status:** Other material. **Occurrence:** recordedBy: Halil Ibrahimi, Ruzhdi Kuçi, Astrit Bilalli, Milaim Musliu; individualCount: 5; sex: males; **Location:** higherGeography: Europe; waterBody: tributary of Morava e Binçës River, Black Sea basin; country: Kosovo; municipality: Gjilan; locality: Zhegër village; verbatimLocality: streamlet above the village; decimalLatitude: 42.31572; decimalLongitude: 21.53148; **Event:** samplingProtocol: UV light trap; samplingEffort: overnight; year: 2017; month: 7; day: 14; fieldNotes: collected with ultraviolet light over the white pan operating from dusk until the next morning; **Record Level:** institutionCode: University of Prishtina "Hasan Prishtina", Faculty of Mathematics and Natural Sciences, Department of Biology; collectionCode: caddisflies of Karadak
- **Type status:** Other material. **Occurrence:** recordedBy: Halil Ibrahimi, Ruzhdi Kuçi, Astrit Bilalli, Milaim Musliu; individualCount: 5; sex: males; **Location:** higherGeography: Europe; waterBody: tributary of Morava e Binçës River, Black Sea Basin; country: Kosovo; municipality: Viti; locality: Letinicë; verbatimLocality: stream above the village; decimalLatitude: 42.28727; decimalLongitude: 21.45736; **Event:** samplingProtocol: UV light trap; samplingEffort: overnight; year: 2017; month: 7; day: 20; fieldNotes: collected with ultraviolet light over the white pan operating from dusk until the next morning; **Record Level:** institutionCode: University of Prishtina "Hasan Prishtina", Faculty of Mathematics and Natural Sciences, Department of Biology; collectionCode: caddisflies of Karadak
- **Type status:** Other material. **Occurrence:** recordedBy: Halil Ibrahimi, Arben Gashi, Besnik Emërllahu, Naman Sinani; individualCount: 4; sex: males; lifeStage: adult; **Location:** higherGeography: Europe; waterBody: tributary of Lumbardhi i Pejës River, Adriatic Sea basin; country: Kosovo; municipality: Pejë; locality: Drelaj; verbatimLocality: stream above the village; decimalLatitude: 42.706667; decimalLongitude: 21.18056; **Event:** samplingProtocol: UV light trap; samplingEffort: overnight; year: 2014; month: 7; day: 14; fieldNotes: collected with ultraviolet light over the white pan operating from dusk until the next morning; **Record Level:** institutionCode: University of Prishtina "Hasan Prishtina", Faculty of Mathematics and Natural Sciences, Department of Biology; collectionCode: caddisflies of Bjeshkët e Nemuna
- **Type status:** Other material. **Occurrence:** recordedBy: Halil Ibrahimi, Arben Gashi, Besnik Emërllahu, Naman Sinani; individualCount: 3; sex: males; lifeStage: adult; **Location:** higherGeography: Europe; waterBody: tributary of Lumbardhi i Pejës River, Adriatic Sea basin; country: Kosovo; municipality: Pejë; locality: Pepaj; verbatimLocality: stream above the village; decimalLatitude: 42.700278; decimalLongitude: 20.143889; **Event:** samplingProtocol: UV light trap; samplingEffort: overnight; year: 2014; month: 9; day: 17; fieldNotes: collected with ultraviolet light over the white pan operating from dusk until the next morning; **Record Level:** institutionCode: University of Prishtina "Hasan Prishtina", Faculty of Mathematics and Natural Sciences, Department of Biology; collectionCode: caddisflies of Bjeshkët e Nemuna

#### Distribution

Slovenia, Italy, Bosnia and Herzegovina and Kosovo (Malicky 2004, Neu et al. 2018, Stanić-Koštroman 2009, Valle 2001).

#### Ecology

We found that the flight period of this subspecies is from May to September. The subspecies is present at different altitudes, from 620 m up to 1307 m.

#### Taxon discussion

The shape of male genitalia and especially of parts which are important for subspecies identification (length, shape and curvature of intermediate appendages, shape and size of dorsal and ventral lobes of inferior appendages and shape of inner basal projections) clearly point to the *slovenica* subspecies (Malicky 2004).

#### Notes

Other species associated with *Polycentropus ierapetra slovenica* in Dërmjak on 12.06.2017 are: *Rhyacophila fasciata* Hagen, 1859 (4 males), *Rhyacophila loxias* Schmid, 1970 (11 males, 2 females), *Rhyacophila polonica* McLachlan, 1879 (7 males and 1 female), (21 males and 2 females), *Rhyacophila tristis* Pictet, 1834 (2 females), (3 males, 2 females), *Glossosoma conformis* Neboiss, 1963 (4 males), *Synagapetus iridipennis* McLachlan, 1879 (1 male); *Philopotamus montanus* (Donovan, 1813) (2 males, 2 females), *Plectrocnemia conspersa* (Curtis, 1834) (2 males), *Polycentropus excicus* Klapálek 1894 (1 male), *Polycentropus flavomaculatus* (Pictet, 1834) (1 male, 2 females), *Psychomyia klapaleki* Malicky, 1995 (1 male, 3 females), *Psychomyia pusilla* (Fabricius, 1781) (5 males, 3 females), *Lype reducta* (Hagen, 1868) (1 male), *Tinodes rostocki* McLachlan, 1878 (1 male), *Tinodes unicolor* (Pictet, 1834) (1 male), *Potamophylax luctuosus* (Piller & Mitterpacher, 1783) (2 males), *Silo graellsii* Pictet, 1865 (12 males, 15 females) and *Oecismus monedula* (Hagen, 1859) (8 males, 3 females).

Other species associated with *Polycentropus ierapetra slovenica* in Zhegër on 14.07.2017 are: *Rhyacophila fasciata* Hagen, 1859 (4 males), *Rhyacophila tristis* Pictet, 1834 (2 males, 2 females), *Philopotamus montanus* (Donovan, 1813) (1 male), *Hydropsyche fulvipes* Curtis, 1834 (2 males); *Hydropsyche saxonica* McLachlan, 1884 (5 males) and *Oecismus monedula* (Hagen, 1859) (2 males).

Other species associated with *Polycentropus ierapetra slovenica* in Letnicë on 20.07.2017 are: *Rhyacophila fasciata* Hagen, 1859 (3 males), *Philopotamus montanus* (Donovan, 1813) (2 males) and *Hydropsyche instabilis* (Curtis, 1834) (4 males) (12 males).

Other species associated with *Polycentropus ierapetra slovenica* in Drelaj on 14.07.2014 are: *Plectrocnemia mojkovacensis* Malicky, 1982 (1 male), *Limnephilus sparsus* Curtis, 1834 (1 male) and *Micropterna sequax* McLachlan, 1875 (1 male).

Other species associated with *Polycentropus ierapetra slovenica* in Pepa on 17.09.2014 are: *Rhyacophila armeniaca* Guerin-Meneville, 1843 (3 males, 2 females), *Rhyacophila palmeni* McLachlan 1879 (2 males) and *Ecclisopteryx keroveci* Previšić, Graf & Vitecek (1 male, 3 females).

### Rhyacophila aurata

Brauer, 1857

3377AAF6-494F-57FA-B5C3-6E2762B7B25F

https://www.gbif.org/species/1433826

#### Materials

- **Type status:** Other material. **Occurrence:** recordedBy: Halil Ibrahimi, Naman Sinani, Arben Gashi; individualID: 1; sex: male; lifeStage: adult; **Location:** continent: Europe; waterBody: tributary of Lumbardhi i Pejës River, Adriatic Sea basin; country: Kosovo; municipality: Pejë; locality: Stankaj; verbatimLocality: streamlet below the village; decimalLatitude: 42.700278; decimalLongitude: 20.143889; **Event:** samplingProtocol: UV light trap; year: 2014; month: 07; day: 20; fieldNotes: collected with entomological net; **Record Level:** institutionCode: University of Prishtina "Hasan Prishtina", Faculty of Mathematics and Natural Sciences, Department of Biology; collectionCode: caddisflies Bjeshkët e Nemuna

#### Distribution

Austria, Bosnia and Herzegovina, Czech Republic, Croatia, France, Germany, Italy, Liechtenstein, Poland, Slovakia, Slovenia, Switzerland (Malicky 2019, Neu et al. 2018).

#### Notes

Other species associated with *Rhyacophila aurata* in Stankaj on 20.07.2014 are: *Rhyacophila loxias* Schmid, 970 (11 males), *Rhyacophila polonica* McLachlan, 1879 (1 male, 1 female), *Rhyacophila palmeni* McLachlan, 1879 (2 males, 1 female), *Rhyacophila tristis* Pictet, 1834 (7 males, 1 female), *Rhyacophila mocsaryi* Klapalek, 1898 (1 female), *Philopotamus montanus* (Donovan, 1813) (1 male) and *Micrasema sericeum* Klapalek, 1902 (1 female).

## Discussion

One species of the Rhyacophilidae family is reported for the first time and one subspecies of Polycentropodidae is documented for the first time with the exact data from the Republic of Kosovo: *Rhyacophila aurata* and *Polycentropus ierapetra slovenica*.

The species *Polycentropus ierapetra* is endemic to South-eastern Europe and Turkey, with several known subspecies which are narrow endemics of certain areas (Malicky 2019, Neu et al. 2018). Currently, the highest-known number of subspecies is from Turkey (*Polycentropus ierapetra anatolica* Sipahiler, 1989, *Polycentropus ierapetra isparta* Sipahiler, 1996, *Polycentropus ierapetra adana* Sipahiler, 1996, *Polycentropus ierapetra milikuri* Malicky, 1975 and *Polycentropus ierapetra baroukus* Botosaneanu and Dia, 1983) and Greece (*Polycentropus ierapetra ierapetra*, *Polycentropus ierapetra ikaria* Malicky, 1974, *Polycentropus ierapetra dirfis* Malicky, 1974 and *Polycentropus ierapetra kalliope* Malicky, 1976). In Bulgaria, only one subspecies (*Polycentropus ierapetra septentrionalis* Kumanski, 1986) is known. The only subspecies with wider distribution is *Polycentropus ierapetra slovenica*, which was described from Slovenia but was later found in Italy and Bosnia and Herzegovina as well (Ibrahimi et al. 2014, Stanić-Koštroman 2009, Valle 2001). During 2014, a single female specimen of *Polycentropus ierapetra* was found in Kosovo (Ibrahimi et al. 2014), but it was impossible to identify up to the subspecies level, due to the fact that females of all subspecies are similar. During the current investigation, we found a large number of males in several localities in Bjeshkët e Nemuna and Karadak mountains and were able to identify them as *Polycentropus ierapetra slovenica*. Considering the narrow distribution of other subspecies, this was somehow unexpected. Our findings make this subspecies one of the most widely distributed. Currently, our findings are the southernmost records of this subspecies. At the same time, these findings are the first reported with exact data for this subspecies for Ecoregion 6. It has been reported by Neu et al. 2018 as present at a single locality in Bjeshkët e Nemuna and here we give exact locality data for several other localities in this area and Karadak Mountains as well.

*Rhyacophila aurata* was found during this investigation in one locality only in Bjeshkët e Nemuna National Park and with only one specimen. This species, although present in many countries in Europe, it is not found frequently in Western Balkans and is absent from neighbouring countries of Kosovo (Malicky 2019, Neu et al. 2018). The species is quite abundant in Central Europe and becomes scarcer towards the Balkan Peninsula. In South-eastern Europe, it is present in numerous localities in Slovenia and Croatia and in only one locality in Bosnia and Herzegovina (Neu et al. 2018) Finding this species during this investigation greatly enlarges its known distribution area.

The number of *Rhyacophila* species has now been increased to fifteen and the number of Polycentropodidae species to ten in the Republic of Kosovo.

Finding of several other rare species during this investigation (such as: *Rhyacophila loxias*, *Rhyacophila palmeni, Polycentropus excicus*, *Plectrocnemia mojkovacensis, Psychomyia klapaleki*, amongst others) greatly enlarges their known distribution area. Previously, they have been reported from only few localities in the Balkans. It is only the second time that *Psychomyia klapaleki* and the third time that *Plectrocnemia mojkovacensis* have been reported from Kosovo. This investigation shows that both mountainous areas where this investigation was conducted harbour a collection of rare caddisfly taxa, many of which are known only from a limited number of localities in the area, based on current knowledge. Further investigations of this area will most certainly increase the number of known species in Kosovo and improve the knowledge about this order of insects in the Balkans.

## Supplementary Material

XML Treatment for Polycentropus ierapetra slovenica

XML Treatment for Rhyacophila aurata

## Figures and Tables

**Table 1.** Details of the six sampling stations in Karadak (S1, S2, S3) and Bjeshkët e Nemuna (S4, S5, S6) mountains in Kosovo.

**Localities**	**Latitude °N**	**Longitude °E**	**Altitude m**
Dërmjak	42.17264	21.31582	620
Letnicë	42.28727	21.45736	625
Zhegër	42.31572	21.53148	640
Stankaj	42.720278	20.048611	1307
Drelaj	42.706667	20.118056	1072
Pepaj	42.700278	20.143889	968
